# Research Progress on the Antiemetic Effect of Traditional Chinese Medicine Against Chemotherapy-Induced Nausea and Vomiting: A Review

**DOI:** 10.3389/fphar.2021.790784

**Published:** 2022-02-09

**Authors:** Yao-Zhong Zhao, Yong-Zhao Dai, Ke Nie

**Affiliations:** School of Chinese Materia Medica, Guangdong Pharmaceutical University, Guangzhou, China

**Keywords:** chemotherapy, nausea, vomiting, traditional Chinese medicine, review

## Abstract

Chemotherapy-induced nausea and vomiting (CINV), a common side effect in antineoplastic treatment, dramatically decreases the quality of life as well as the compliance of cancer patients. Although numerous antiemetic agents have been used for CINV treatment, its adverse reactions as well as its inadequate control toward delayed emesis still limit its clinical usage. Traditional Chinese medicine (TCM), with more than 3,000 years of practical history in Asia, has been successfully applied to mitigate chemotherapy-induced side effects. Growing attention is drawn to the antiemetic effect of TCM against CINV due to its promising therapeutic property and higher safety recently. In this review, we summarize the classic antiemetic TCM-based treatment and its mechanisms, so as to provide a theoretical basis for further investigations of TCM against CINV in the future.

## 1 Introduction

Chemotherapy-induced nausea and vomiting (CINV) is a common treatment-related side effect in cancer patients receiving antineoplastic treatment (Navari and Aapro, 2016). Nausea and vomiting, one of the greatest repugnant emotions on cancer patients during chemotherapy treatment, dramatically decreased the quality of life as well as the compliance of cancer patients (Hesketh, 2008). In the past decades, ever since dopamine receptor antagonists, such as metoclopramide, were approved for highly emetogenic agent-induced emesis, great advances have been made in the development of antiemetic medicines against CINV (Hesketh et al., 1997; Roila et al., 2006). A growing number of novel antiemetic drugs, such as 5-hydroxytryptamine type 3 receptor (5-HT_3_R) antagonist ondansetron, neurokinin-1 receptor (NK-1R) antagonist aprepitant, and others are successively recommended as standard prophylaxis for CINV clinically (Roila et al., 2010; Berger et al., 2017). At present, the combination of 5-HT_3_R antagonist, NK-1R antagonist, and dexamethasone is the standard antiemetic protocol of CINV (Razvi et al., 2019). Despite visible progress, side effects of these antiemetic agents itself still limit their clinical usage. The administration of 5-HT_3_R antagonist is commonly accompanied by headache and constipation (Schwartzberg et al., 2014), and the NK-1R antagonist treatment is usually associated with asthenia, fatigue, and hiccups (Hesketh et al., 2003). Thus, there is an urgent need in searching for new antiemetic agents with promising antiemetic property and few side effects.

Traditional Chinese medicine (TCM), with more than 3,000 years of clinical practice history in Asia, is able to effectively treat CINV. For instance, ginger (*Zingiber officinale* Roscoe), a traditional Chinese herb, has a promising antiemetic effect in treating chemotherapy-induced emesis (Grant and Lutz, 2000; Pillai et al., 2011; Konmun et al., 2017). Likewise, Liu-Jun-Zi decoction (LJZD), also known as “Rikkunshito” in Japanese medicines (kampo) and “Yukgunja-Tang” in Korea, is able to alleviate chemotherapy-induced side effects like nausea, emesis, and anorexia (Ohnishi et al., 2017). The literature suggests that TCM has a wide considerable future and unique advantages in preventing and treating CINV compared with the existing antiemetic medicines. On one hand, unlike chemical drugs that only intervene in a single target, TCM has multiple components and interacts with multiple targets in treating diseases. For example, ginger and its active ingredients like gingerols are shown to prominently exert antiemetic effect by blocking 5-HT_3_R, NK-1R, and D_2_R as well as regulating gastrointestinal dysfunction (Cheng et al., 2020; Tian et al., 2020). On the other hand, combining Xiao-Ban-Xia decoction (XBXD) and the 5-HT_3_R antagonist as the prophylaxis of CINV clinically not only increases the antiemetic efficacy on delayed vomiting but also reduces the adverse reactions of the 5-HT_3_R antagonist (Ouyang et al., 2002; Liu, 2018). Also, the relatively low price of Chinese herbal medicine may reduce the economic burden of cancer patients to a certain extent, compared with current antiemetic agents. In this review, we take a retrospection on the mechanism of CINV and summarize the classic antiemetic TCM-based treatment and its potential mechanism to provide a theoretical basis for further investigations of TCM against CINV in the future.

## 2 Pathological Mechanism of Chemotherapy-Induced Nausea and Vomiting

### 2.1 Types of Chemotherapy-Induced Nausea and Vomiting

Generally speaking, CINV can be divided into five types, including acute, delayed, breakthrough, anticipatory, and refractory CINV, based on the occurrence time of nausea and vomiting as well as the patient’s responses after chemotherapeutic treatments (Navari and Aapro, 2016) (Table 1). The acute phase of CINV refers to the emesis that occurs within the first 24 h after chemotherapy (Hesketh et al., 1997), and emesis from 24 h to several days (day 2 to day 5 post chemotherapy) is defined as delayed CINV (Bloechl-Daum et al., 2006). Breakthrough CINV happens when emesis occurs in patients despite receiving appropriate prophylactic treatments (Navari, 2015). Due to the memory of an adverse experience during previous chemotherapy, patients have formed a conditioned response to occurrence of emesis before the next chemotherapy cycles begin, which could be defined as anticipatory CINV (Morrow et al., 1998). Refractory CINV refers to emesis occurring in subsequent chemotherapy due to the failure to prevent nausea and/or vomiting by using antiemetic medicines in earlier chemotherapy cycles (Navari, 2015).

**TABLE 1:** Types of CINV.

Type of CINV	Feature	References
Acute	Emesis occurs within the first 24 h after chemotherapy	Hesketh et al. (1997)
Delayed	Emesis occurs from 24 h to several days after chemotherapy	Bloechl-Daum et al. (2006)
Breakthrough	Emesis occurs despite receiving appropriate prophylactic treatments	Navari (2015)
Anticipatory	Emesis occurs before the next chemotherapy cycles begin because of an adverse memory during previous chemotherapy	Morrow et al. (1998)
Refractory	Emesis occurs due to prevention failure by antiemetic agents	Navari (2015)

### 2.2 Pathological Mechanism of Chemotherapy-Induced Nausea and Vomiting

Despite the research progress made on the pathological mechanism of CINV, the underlying mechanism still has not been fully clarified. To date, several neurotransmitters, including 5-hydroxytryptamine (5-HT), substance P (SP), dopamine (DA), and others, are confirmed as key meditators of CINV (Hesketh, 2008). Early published studies found that blocking central and peripheral dopamine type 2 receptor (D_2_R) *via* D_2_R antagonist metoclopramide was a feasible method against mildly or moderately emetogenic chemotherapy-induced emesis (Chevallier et al., 1997; Jordan et al., 2005). Then, 5-HT_3_R and NK-1R started to draw attention. In the peripheral nervous system, it was discovered that chemotherapeutic agents stimulated intestinal enterochromaffin cells to release 5-HT and activated the 5-HT_3_R in the terminal ends of the vagal afferents. Then, the fibers transmitted impulse to the center, which induced emetic reflex and sent efferent signals toward the chemoreceptor trigger zone and vomiting center and eventually induced acute emesis (Hesketh, 2008). As for the central nervous system, the chemotherapeutic drugs stimulated the synthesis and release of SP and activated NK-1R located at the area postrema and nucleus tractus solitarius to induce delayed nausea and/or vomiting (Hargreaves et al., 2011). The latest research identified growth differentiation factor 15 (GDF-15) to be a novel meditator that might induce nausea, emesis, and anorexia (Borner et al., 2020a). The reports published by Borner et al. (2020b) and Breen et al. (2020) offered a new pathological mechanism of CINV. According to their studies, the administration of chemotherapeutic agents significantly increased the circulating GDF-15 levels in the cisplatin-induced pica model of rats as well as the nonhuman primates and induced nausea, vomiting, and anorexia *via* activating the area postrema glial-derived neurotrophic factor (GDNF) family receptor α-like (GFRAL).

**FIGURE 1:**
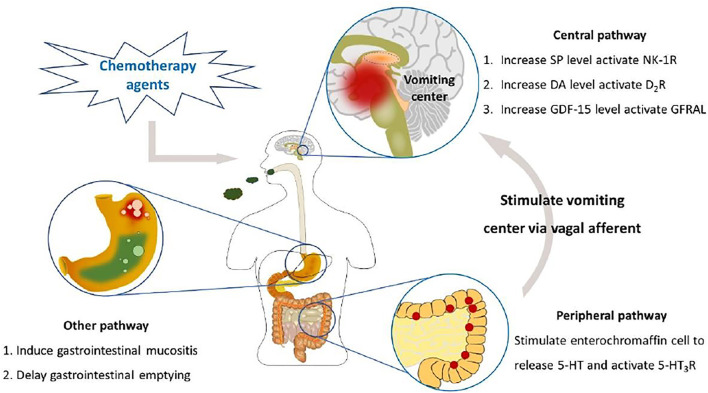
The mechanism of CINV. In the peripheral nervous system, chemotherapy drugs stimulate enterochromaffin cells to release 5-HT, which activates 5-HT_3_R and then stimulates the vomiting center via the vagal afferent, eventually inducing emesis. In the central nervous system, the increased levels of SP, DA, and GDF-15 induce vomiting via activating their respective receptors. Also, chemotherapy drugs induce gastrointestinal mucositis and delayed gastric emptying, which may be the other potential mechanism that leads to nausea and vomiting.

Furthermore, chemotherapy-induced delayed gastric emptying and gastrointestinal mucositis also play an important role in the pathology of CINV (Marx et al., 2017). Chemotherapeutic agents, like cisplatin and doxorubicin, delay gastric emptying, while the classic CINV prophylaxis medicines, such as granisetron and ondansetron, promote gastrointestinal motility, indicating that gastrointestinal dysfunction is closely related to the onset of CINV (Ando et al., 2012; Vera et al., 2014). Besides, chemotherapeutic drugs induce nausea and vomiting and promote the release of reactive oxygen species (ROS), which activates the inflammatory signaling pathway and upregulates the expression of cytokines to induce gastrointestinal mucositis, suggesting an underlying relationship between CINV and gastrointestinal mucositis (Sonis, 2004; Logan et al., 2008). Indeed, many antiemetic medicines are demonstrated to alleviate chemotherapy-related gastrointestinal mucositis, and analogously, anti-inflammatory drugs can also be used to treat emesis (Sam et al., 2001; Girod et al., 2002; Yasuda et al., 2013). The administration of ramosetron and ondansetron, the classic 5-HT_3_R antagonist antiemetic medicines, ameliorates 5-fluorouracil-induced intestinal mucositis via downregulation of the expression of small intestine proinflammatory cytokines, including tumor necrosis factor alpha (TNF-α), interleukin 1 beta (IL-1β), interleukin 6 (IL-6), and interleukin 18 (IL-18), and suppresses apoptosis in murine intestinal crypt cells (Yasuda et al., 2013). Dexamethasone, the common glucocorticoid anti-inflammatory agent, has the property to relieve cisplatin-induced emesis and is recommended by antiemetic guidelines as an adjuvant therapy of CINV, which might be due to its anti-inflammatory effect (Sam et al., 2001; Roila et al., 2010; Berger et al., 2017). Homogeneously, the nonsteroidal anti-inflammatory drug indomethacin also has antiemetic property in treating cisplatin-induced emesis (Girod et al., 2002). The results shown above suggest that gastrointestinal motility disorder and mucositis might play a vital role in the mechanism of chemotherapy-induced emesis. The pathological mechanism of CINV is shown in Figure 1.

## 3 Antiemetic Effect of Traditional Chinese Medicine

### 3.1 Xiao-Ban-Xia Decoction

XBXD, an antiemetic traditional Chinese formula, recorded in the prescription of the *Golden Chamber* by Zhang Zhongjing in the Han dynasty, is composed of *Pinellia ternata* (Thunb.) Makino and *Zingiber officinale* Roscoe. The antiemetic effects of XBXD against CINV have been confirmed by a large number of clinical studies conducted in Chinese (Ouyang et al., 2002; Liu, 2018; Leng and Li, 2020). Results showed that combining XBXD and the 5-HT_3_R antagonist as the prophylaxis of CINV clinically not only increases the antiemetic efficacy on delayed vomiting but also reduces the adverse reactions of the 5-HT_3_R antagonist. In preclinical studies, the antiemetic efficacy of XBXD against CINV has been demonstrated in several animal models, including vomiting model of minks, pica model of rats, and others (Qian et al., 2010a; Qian et al., 2011; Meng et al., 2020). In the non-emetic model of rodents, the consumption of nonnutritive substances such as kaolin (i.e., pica behavior) is analogous to emesis in humans (Takeda et al., 1993). Since this cluster of findings, numerous reports have investigated the potential mechanisms of XBXD against CINV.

The earlier proposed antiemetic mechanism of XBXD related to the reduction of 5-HT level and blockade of the 5-HT_3_R in the ileum (Wang et al., 2010). It was further indicated that XBXD pronouncedly ameliorated CINV *via* markedly reducing the tryptophan hydroxylase (TPH) level, upregulating the expression of monoamine oxidase A (MAO-A) and serotonin reuptake transporter (SERT) mRNA (Yu et al., 2015a; Li et al., 2020). TPH is a rate-limiting enzyme of 5-HT synthesis, and the MAO-A and SERT are a vital transshipment metabolic approach of 5-HT (Gershon and Tack, 2007; Rahman et al., 2011; Naoi et al., 2018). Results suggested that the potential mechanisms of XBXD against CINV may in large part be due to the regulation of 5-HT synthesis and metabolism disorders and the blockade of 5-HT_3_R. Besides, XBXD significantly inhibited tyrosine hydroxylase (TH), a synthetic rate-limiting enzyme of DA (Daubner et al., 2011), to reduce the overexpression of DA and block the peripheral D_2_R, which may be another antiemetic mechanism of XBXD (Yu et al., 2015b; Yu, 2015). The abnormally high level of SP and the activation of its related receptor may be the main reasons for delayed vomiting. Recent studies found that XBXD and Xiao-Ban-Xia-Fu-Ling decoction (XBFD, adding *Poria cocos* (Schw.) Wolf on the basis of XBXD) markedly inhibited pre-protachykinin-A (PPT-A) mRNA, which is the precursor of SP synthesis expression, in medulla oblongata and gastric antrum (Harrison and Geppetti, 2001). Also, XBXD and XBFD blocked the NK-1R in both the medulla oblongata and gastric antrum, which might contribute to its antiemetic effect on delayed CINV (Qian et al., 2010a; Zang et al., 2011).

Besides, in the cisplatin-induced pica model of rats, pretreatment with XBXD significantly reduced the consumption of kaolin and alleviated gastrointestinal mucositis (Li et al., 2017; Du et al., 2018; Meng et al., 2020). It was suggested that the protection of XBXD on the gastrointestinal mucous membrane was due in large part to the inhibition of the activation of ileum nuclear factor-kappa B (NF-κB), cyclooxygenase-2 (COX-2), and NOD-like receptor family pyrin domain containing 3 (NLRP3) inflammasome, which might contribute to the antiemetic effect of XBXD against CINV. By using RNA sequencing, a study further supported that the antiemetic effect of XBXD was closely related to its anti-inflammatory property in the cisplatin-induced pica model of rats (Li et al., 2020). With XBXD treatment, the pica behavior was markedly alleviated; the expression of ileum proinflammatory genes like *Tnf*, *Il1b*, *Nlrp3*, and *Peg12* was downregulated; and the activation of inflammation-related pathways like NF-κB, interleukin 17 (IL-17), and the NOD-like receptor signaling pathway was significantly inhibited compared with the model. Furthermore, it is worth noting that XBXD effectively regulates chemotherapy-induced delayed gastric emptying. Previous studies demonstrated that XBXD promoted gastric emptying and intestinal movement in cisplatin-treated mice (Liu et al., 2017). Further investigation was carried out on the cisplatin-induced pica model of rats; pretreatment with XBXD ameliorated cisplatin-induced abnormal gastrointestinal hormone expression, such as gastrin, motilin, somatostatin, and vasoactive peptide, suggesting that the regulation of the gastrointestinal function might also be a potential antiemetic mechanism of XBXD against CINV (Zhang et al., 2015). These studies seem to provide an interesting insight on the inflammatory and gastrointestinal functions of XBXD against CINV; however, improvements should be made in future studies. Firstly, as for the grouping of experimental animals, appropriate anti-inflammatory agents or inflammatory pathway inhibitors should be added, in order to further validate the correlation between emesis and gastrointestinal mucositis. In addition, appropriate gastrointestinal cell lines such as intestinal epithelial cells (Wang et al., 2021) should also be selected to investigate the anti-inflammatory effects of XBXD.

**FIGURE 2:**
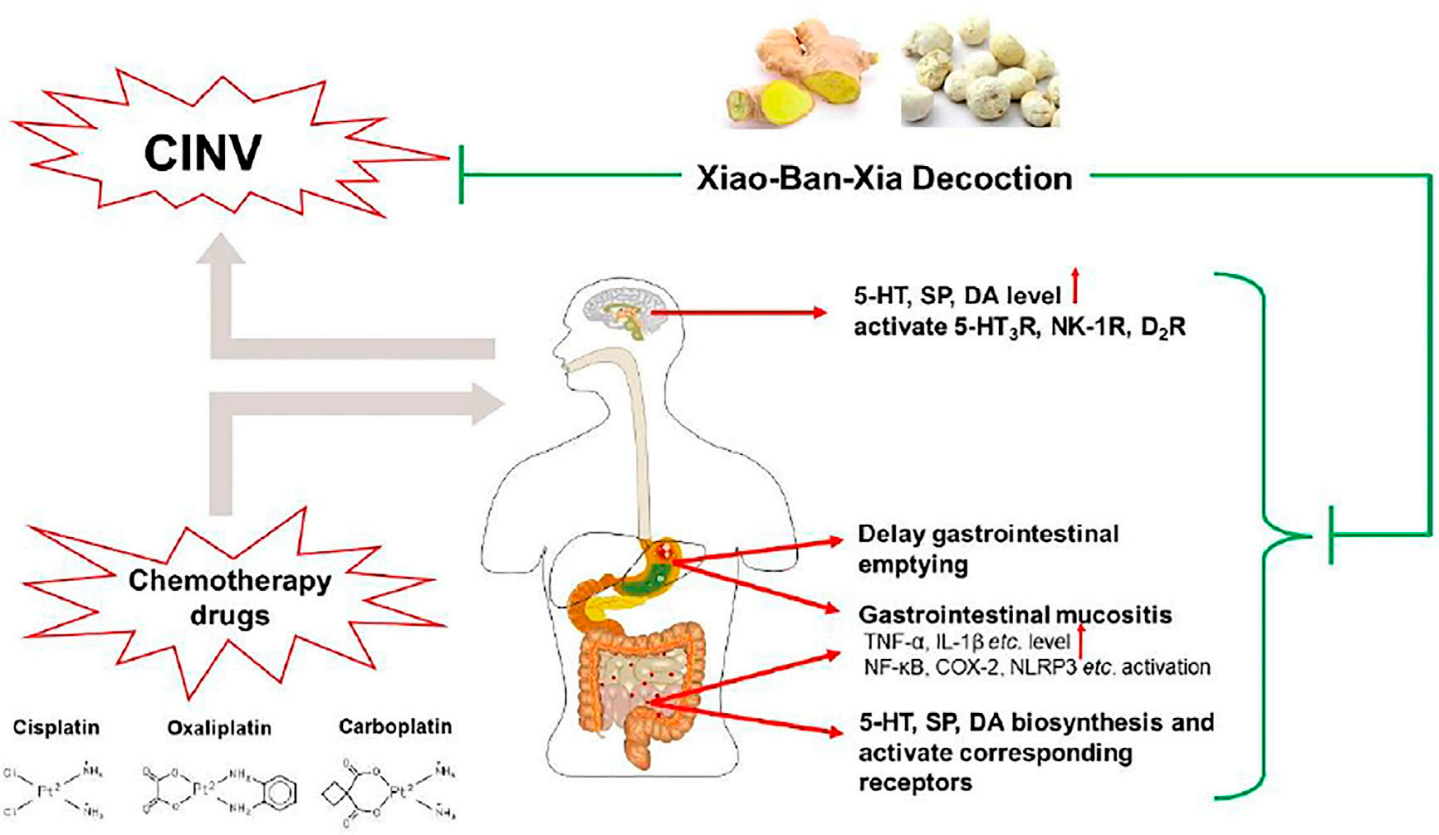
The antiemetic mechanism of XBXD against CINV. In the central nervous system, XBXD markedly reduces levels of 5-HT, SP, and DA and blocks 5-HT_3_R, NK-1R, and D_2_R. In the peripheral nervous system, XBXD inhibits the biosynthesis of 5-HT, SP, and DA and blocks their respective receptors. Also, XBXD significantly mitigates chemotherapy-drug-induced gastrointestinal mucositis and delayed gastric emptying.

Taken together, XBXD acts on different pathways against CINV, including blocking the 5-HT_3_R, NK-1R, and D_2_R; ameliorating gastrointestinal inflammation; and regulating the gastrointestinal motility disorders (Figure 2). Indeed, the effect of XBXD on treating CINV seems promising; however, further evaluation on the toxicity of XBXD in the presence of *P. ternata* is still needed. *P. ternata* is a common toxic herbal medicine; its reproductive toxicity, hepatotoxicity, and irritation have been observed (Ji et al., 2014). Interestingly, it is also reported that a clinical dose of *P. ternata* extract is able to treat CINV effectively and safely, similar to ondansetron in humans, and that no other side effects were observed in mice (Tan et al., 2004). As for XBXD, although ginger can reduce its toxicity according to Chinese medical theory, whether the clinical dosage of *P. ternata* in XBXD is safe still needs further data for reference.

### 3.2 Liu-Jun-Zi Decoction

LJZD, recorded in the True Biography of Medicine by Yu Tuan in the Ming dynasty and composed of Panax ginseng C.A. Mey., Atractylodes macrocephala Koidz., Poria cocos (Schw.) Wolf, Glycyrrhiza glabra L., and Citrus × aurantium L., P. ternata (Thunb.) Makino, is a common traditional Chinese formula that has been adapted to treat functional dyspepsia, anorexia, nausea, and emesis with a long history. LJZD has antiemetic property against chemotherapeutic agents inducing nausea and vomiting (Hattori et al., 2013; Morishige, 2017; Yoshiya et al., 2020). Several prospective studies have tested the antiemetic effect of LJZD in preventing CINV clinically. In cancer patients undergoing antineoplastic treatments, it is reported that LJZD significantly ameliorated chemotherapy-induced anorexia as well as nausea (Ohno et al., 2011; Seike et al., 2011). A recent clinical trial proved that pretreatment with LJZD was able to provide additive effect for the prevention of chemotherapy-induced emesis and anorexia (Ohnishi et al., 2017). The underlying mechanism of LJZD against CINV has not been adequately demonstrated yet. The administration of LJZD improved gastric emptying via blocking the 5-HT_3_R pathway, which might be one of the potential mechanisms of LJZD against CINV (Tominaga et al., 2011). Further, ghrelin is an appetite-stimulating or hedonic-eating hormone in the digestive system, which plays a vital role in reducing nausea and vomiting via activating the ghrelin receptor (Chen and Tsai, 2012; Sanger and Furness, 2016). In the cisplatin treatment model of rats, pretreatment with LJZD markedly increased the food intake and plasma ghrelin level via antagonizing 5-hydroxytryptamine type 2B receptor (5-HT_2B_R) and 5-hydroxytryptamine type 2C receptor (5-HT_2C_R), suggesting that the mechanism of LJZD was related to the mitigation of chemotherapy-induced anorexia and emesis by stimulating ghrelin secretion (Takeda et al., 2008; Yakabi et al., 2010).

**FIGURE 3:**
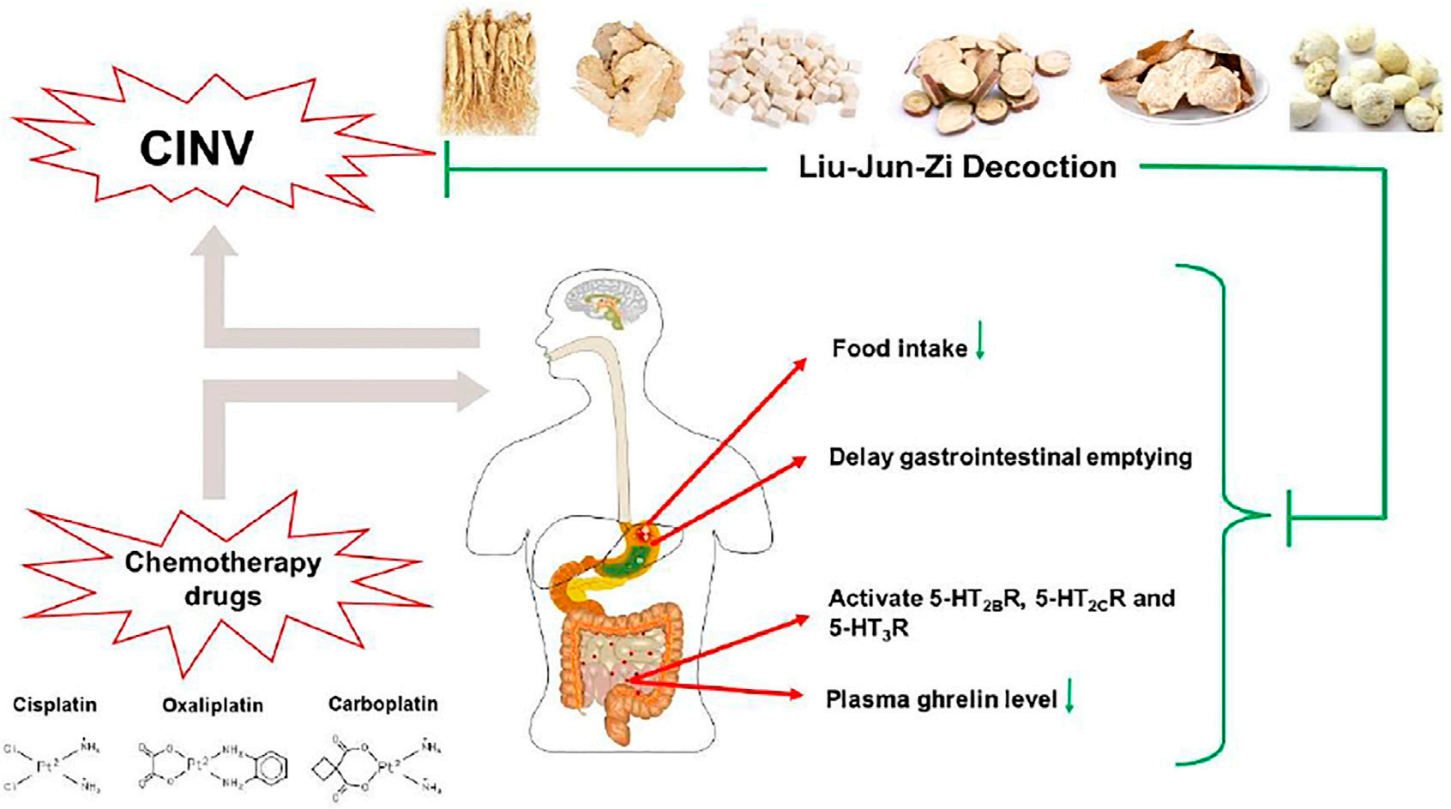
The antiemetic mechanism of LJZD against CINV. LJZD markedly increases the food intake and circling ghrelin level via blocking 5-HT_2B_R and 5-HT_2C_R, Besides, LJZD improves the cisplatin-induced delayed gastric emptying via blocking the 5-HT_3_R pathway.

Taken together, LJZD provides a potential antiemetic effect against CINV, and its underlying mechanisms are partly concerned with the blockade of 5-HT_3_R, 5-HT_2B_R, and 5-HT_2C_R and the increase of the circling ghrelin level (Figure 3). Further studies in more animal models and clinical trials are needed.

### 3.3 Ginger

Ginger (*Zingiber officinale* Roscoe), a traditional herb in Asia and Europe, has been a common spice for various recipes around the world, especially in China and India (Grant and Lutz, 2000). Both clinical and animal studies have proved that ginger can be used as a vital approach in mitigating chemotherapy-induced emesis (Palatty et al., 2013). In clinical studies, the antiemetic effects of ginger and its active compound 6-gingerol in preventing and treating CINV were sufficiently confirmed (Pillai et al., 2011; Ryan et al., 2012; Arslan and Ozdemir, 2015; Konmun et al., 2017; Uthaipaisanwong et al., 2020). It has been reported that pretreatment with ginger and 6-gingerol not only reduced the incident of vomiting but also increased appetite and improved the quality of life in cancer patients. In animal studies, the cytotoxic-drug-induced vomiting model of *Suncus murinus* and dogs both showed that ginger acetone extract possessed an antiemetic effect against CINV (Yamahara et al., 1989; Sharma et al., 1997). Besides, acetone and 50% ethanolic extract of ginger and ginger juice markedly reversed cisplatin-induced delayed gastric emptying (Sharma and Gupta, 1998). Results above showed a potential antiemetic effect of ginger, but further investigation of its potential antiemetic mechanism is needed. On this basis, many published data and ongoing trials are underway, investigating the mechanism of ginger and its active compounds against chemotherapy-induced emesis.

A study reported that ginger and its major pungent constituents (6-gingerol, 6-shogaol, and zingerone) significantly blocked 5-HT-evoked response of the visceral vagal afferent nerve, similar to ondansetron, suggesting a potential antiemetic effect by blocking 5-HT_3_R (Jin et al., 2014). Gingerols, consisting of 6-, 8-, and 10-gingerols, are the major pungent compound of ginger. Recently, several studies on the cisplatin-induced vomiting model of minks (Qian et al., 2009; Qian et al., 2010b; Tian et al., 2020) and pica model of rats (Qian et al., 2016; Tian et al., 2020) showed that the administration of gingerols markedly reduced area postrema and ileum 5-HT, SP, and DA levels via inhibiting their syntheses and promoting their metabolism, as well as blocking 5-HT_3_R, NK-1R, and D_2_R. However, the composition and content of the active compound gingerol were not marked in these studies, which greatly reduced reliability and repeatability. To better evaluate the antiemetic mechanism of ginger, the major monomer 6-gingerol is used as an approach in a cisplatin-induced pica model of rats. It was found that pretreatment with 6-gingerol significantly mitigated CINV through downregulating the synthesis and promoting the metabolism of 5-HT, as well as blocking 5-HT_3_R (Cheng et al., 2020). Moreover, 6-gingerol also ameliorated microbiota disorders to alleviate cisplatin-induced kaolin intake in the pica model of rats, regulated gastrointestinal hormones, and promoted gastric emptying (Feng et al., 2019; Zhang et al., 2021).

**FIGURE 4:**
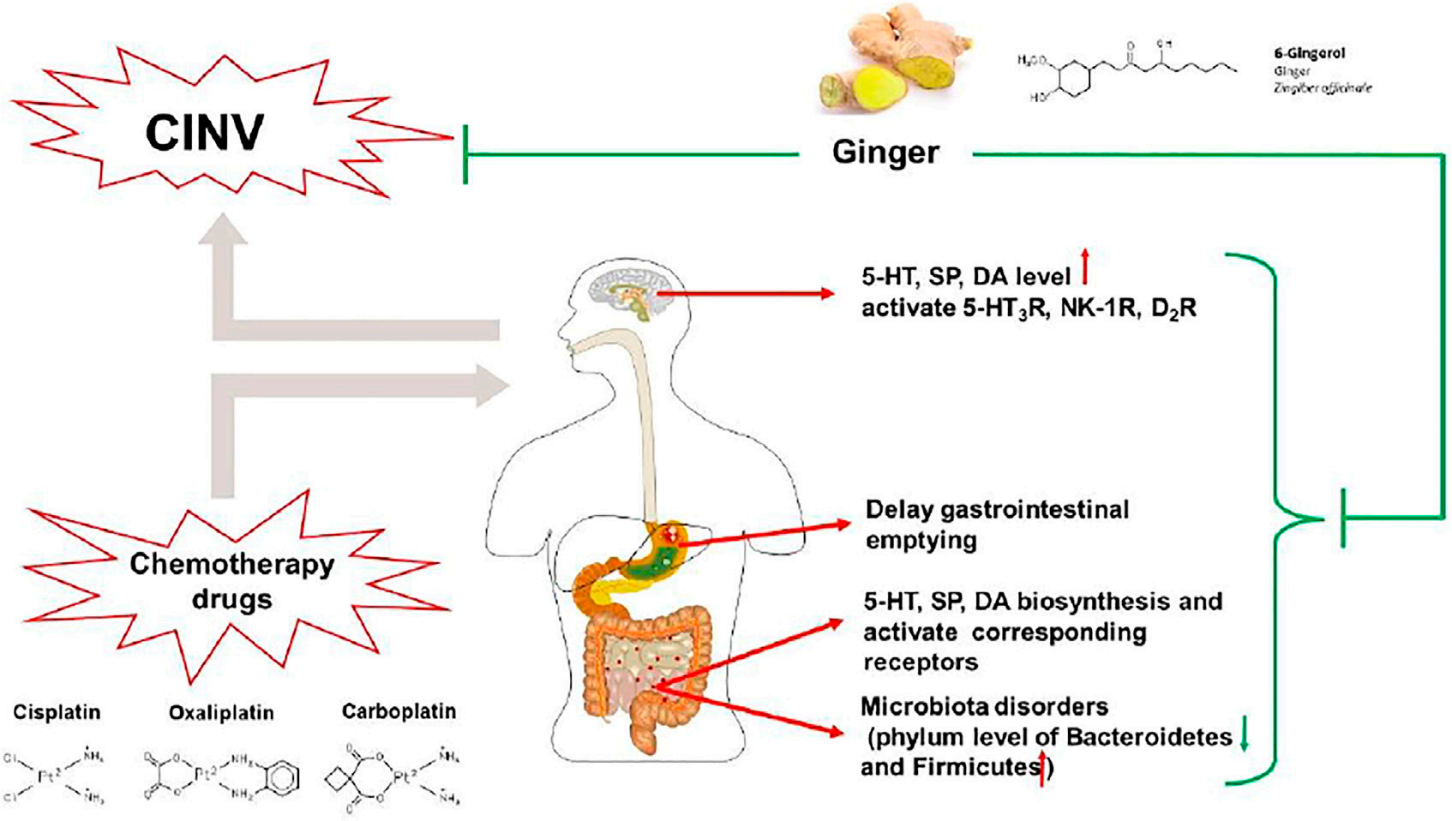
The antiemetic mechanism of ginger against CINV. In the central nervous system, ginger markedly reduces levels of 5-HT, SP, and DA and blocks 5-HT_3_R, NK-1R, and D_2_R. In the peripheral nervous system, ginger inhibits the biosynthesis of 5-HT, SP, and DA, blocking their respective receptors. Also, ginger ameliorated microbiota disorders and delayed gastric emptying induced by chemotherapeutic agents.

Taken together, the potential antiemetic mechanism of ginger against CINV relates to the modulation of 5-HT, SP, and DA signaling pathways and the improvement of gastrointestinal disorders (Figure 4). However, though the antiemetic effect of ginger against CINV has been validated in animal studies, its clinical efficacy is still controversial, which may be related to the different dosages and dosage forms of ginger. Additional studies are warranted to clarify the optimal dosage and dosage forms of ginger. Moreover, several potential antiemetic ingredients such as 6-gingerol have been identified from ginger. Further studies may also focus on its structural modification for antiemetic effect enhancement.

### 3.4 Forsythiae Fructus

*Forsythiae Fructus* (*Forsythia suspensa* (Thunb.) Vahl) is a traditional Chinese herb that is commonly used in clearing heat and detoxification. In Chinese folk medicine, *Forsythiae Fructus* is also applied to treat vomiting induced by multifactorial inducements. Moreover, the antiemetic effect of *Forsythiae Fructus* has been demonstrated in a copper-sulfate-pentahydrate-induced emesis model of frogs (Kinoshita et al., 1996), which laid a foundation for *Forsythiae Fructus* against chemotherapy-induced emesis.

The following studies investigated the antiemetic efficacy and the potential mechanism of *Forsythiae Fructus* against CINV. In a cisplatin-induced pica model of rats, pretreatment with *Forsythiae Fructus* extract markedly reduced plasma 5-HT level and ileum 5-HT_3_R expression, suggesting a potential antiemetic effect mainly related to the blockade of 5-HT/5-HT_3_R (Wang, 2010). Besides, *Forsythiae Fructus* also alleviated gastrointestinal mucosa injuries via inhibiting the activation of NF-κB, COX-2, and NLRP3 inflammasomes and reduced plasma proinflammatory cytokine levels, which may be another antiemetic mechanism of *Forsythiae Fructus* (Meng et al., 2019; Meng et al., 2021). Furthermore, *Forsythiae Fructus* extract administration markedly regulated the gastrointestinal hormones like gastrin, motilin, and somatostatin so as to promote the delayed gastric emptying induced by cisplatin (Zuo, 2015; Zhang et al., 2018). Forsythiaside A, a major active constituent of *Forsythiae Fructus*, had an effect to regulate the gastrointestinal hormones as well as the activity of acetylcholinesterase and nitric oxide synthase in the gastric antrum and ileum, suggesting a similar effect to regulate cisplatin-induced gut motility disorders (Bi et al., 2021).

**FIGURE 5:**
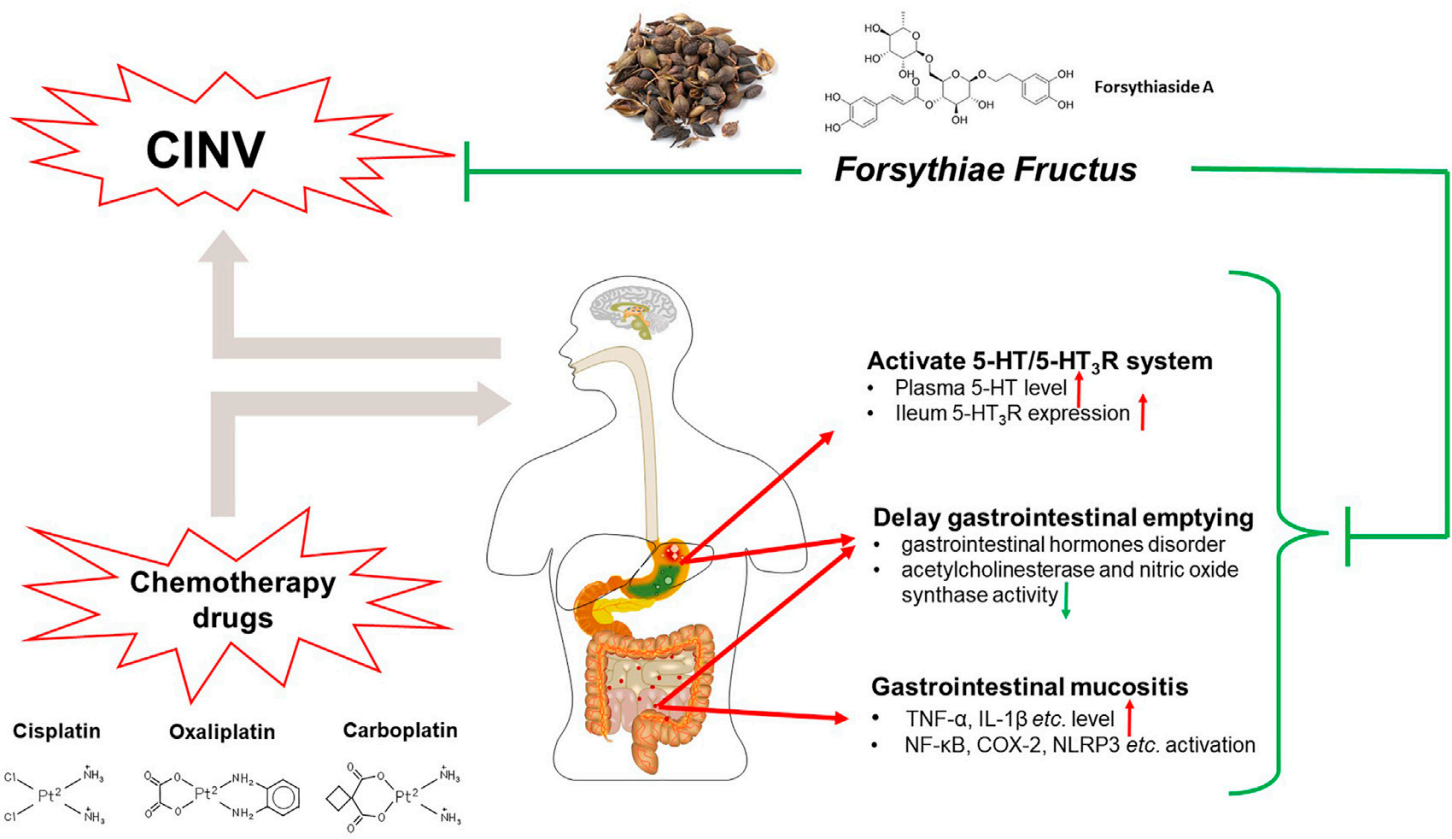
The antiemetic mechanism of *Forsythiae Fructus* against CINV. In the peripheral nervous system, *Forsythiae Fructus* markedly reduces levels of 5-HT and blocks 5-HT_3_R. Besides, *Forsythiae Fructus* significantly mitigates chemotherapy-drug-induced gastrointestinal mucositis and delayed gastric emptying.

The antiemetic mechanisms of *Forsythiae Fructus* against CINV might involve blocking the 5-HT_3_R pathway, alleviating gastrointestinal inflammation, and regulating gastrointestinal motility disorders (Figure 5). Although animal studies have confirmed a promising antiemetic effect of *Forsythiae Fructus*, additional clinical trials are needed to provide evidence before drawing a conclusion.

### 3.5 Ginseng and American Ginseng

Ginseng (*Panax ginseng* C.A. Mey) and its processed herb Red Ginseng, the classic spleen-Qi nourishing Chinese herbal medicine, are good at reinforcing vital energy. Recently, a comparative study conducted by Kim et al. (2005) tested the antiemetic effect of ginseng. In this study, ferrets were treated with cisplatin to establish a nausea-and-vomiting model; pretreatment with Red Ginseng extract significantly reduced vomiting and nausea of ferrets in a dose-dependent manner, suggesting that ginseng might be used as a potential antiemetic agent for CINV treatment. The following studies confirmed the antiemetic effect of ginseng in the cisplatin-induced pica model of rats and identified that both ginseng saponin and non-saponin fractions of ginseng had a significant antiemetic efficacy (Raghavendran et al., 2011; Sathyanath et al., 2013).

American ginseng (*Panax quinquefolius* L.), a close relative species of ginseng, is a traditional herbal medicine in the regions of North America and Asia (Szczuka et al., 2019). In the past decades, several published studies have suggested that American ginseng has an antiemetic effect on cancer patients undergoing chemotherapy (Haniadka et al., 2012). In the cisplatin-induced pica model of rats, it was reported that pretreatment with American ginseng resulted in a significant reduction in kaolin consumption, suggesting an antiemetic effect of American ginseng against CINV (Mehendale et al., 2004). And Mehendale et al. demonstrated that American ginseng berry extract and ginsenoside Re, the antioxidant constituents of American ginseng, might be the potential antiemetic effective parts of American ginseng (Mehendale et al., 2005). An *in vitro* experiment made further elucidation on the antiemetic effect of ginseng saponins, including total saponin, panaxadiol saponin fraction, panaxatriol saponin fraction, ginsenoside Rb1, and ginsenoside Rg1. In a recombinant 5-HT type 3A receptor (5-HT_3A_R)-expressing *Xenopus laevis* oocyte model, all ginseng saponins significantly inhibited the peak current on 5-HT_3A_R induced by 5-HT, suggesting that ginseng saponins have a substantial inhibitory effect on 5-HT_3A_R, which might be the vital mechanism of American ginseng against CINV (Min et al., 2003).

Taken together, the antiemetic effect of ginseng and American ginseng had been confirmed in the pica model of rats and vomiting model of ferrets. But further studies in more animal models and clinical trials are needed.

### 3.6 Scutellaria baicalensis

*Scutellaria baicalensis* (*Scutellaria baicalensis* Georgi) is a traditional Chinese herb that is commonly used for clearing heat and eliminating dampness. Recently, some studies have demonstrated the antiemetic effect of *Scutellaria baicalensis* against CINV. In the cisplatin-induced pica model of rats, Mehendale et al. showed that the approach of *Scutellaria baicalensis* extract markedly reduced the kaolin consumption of rats, suggesting its potential antiemetic effect in treating chemotherapy-induced emesis (Aung et al., 2003; Mehendale et al., 2004). Besides, it was reported that *Scutellaria baicalensis* extract and its constituent baicalein significantly alleviated protease inhibitor ritonavir-induced pica behavior and delayed gastric emptying in rats (Mehendale et al., 2007). Although the antiemetic effect of *Scutellaria baicalensis* has been confirmed in the pica model of rats, its potential antiemetic mechanism is still obscure. Thus, further studies should be focused on investigating its related mechanism. Also, relevant clinical trials may also be carried out to comprehensively evaluate the antiemetic effect of *Scutellaria baicalensis*.

### 3.7 Ganoderma lucidum

*Ganoderma lucidum* (*Ganoderma lucidum* (Leyss. ExFr.) Karst.), a common tonic Chinese herbal medicine, has shown immunity enhancement and antineoplastic efficacy. A recent study in a cisplatin-induced pica model of rats showed a promising antiemetic effect of *Ganoderma lucidum* against CINV (Wang et al., 2005a). Also, *Ganoderma lucidum* polysaccharides significantly inhibited cisplatin-induced pica behavior in mice, and the inhibition efficacy is better than that of granisetron (Yan, 2008). Despite the performance of animal studies, further investigation of its antiemetic mechanism and clinical antiemetic efficacy is needed.

### 3.8 Grape Seed

Grape seeds (*Vitis vinifera* L.) used to be a waste product of the grape processing industry, and yet recent studies suggest its potential antiemetic effect on CINV prevention. Wang et al. (2005b) had confirmed the antiemetic effect of grape-seed extract, a widely used antioxidant dietary supplement, in a cisplatin-induced pica model of rats. In this study, three types of grape-seed extract had been tested, with different percentage compositions of the five major antioxidant constituents, namely, gallic acid, catechin, epicatechin, procyanidin B2, and epicatechin gallate. Administration of all three grape-seed extracts reduced kaolin consumption compared with cisplatin treatment in rats in varying degrees, suggesting that the appropriate dose of grape-seed extracts alleviated cisplatin-induced emesis. Later, Alam et al. conducted an experiment on a cisplatin-induced emesis model of pigeons to test the antiemetic effect of grape-seed proanthocyanin, an antioxidant constituent (Alam et al., 2017). The result showed that administration of grape-seed proanthocyanin in pigeons markedly reduced the vomiting bouts, retching rate, and weight loss rate compared with cisplatin treatment, suggesting a potential antiemetic mechanism of grape seeds in preventing CINV, which may in large part be due to its antioxidative property.

### 3.9 Rhus verniciflua

*Rhus verniciflua* (*Toxicodendron vernicifluum* (Stokes) F.A. Barkley) is a traditional herbal medicine to treat gastrointestinal dysfunction. Recent studies have reported its potential antiemetic effect in a cisplatin-induced pica model of rats (Kim et al., 2017). Results showed that pretreatment with *Rhus verniciflua* extract has a significant effect on reducing the consumption of kaolin in both the acute and delayed phases, and the potential mechanism of *Rhus verniciflua* extract against CINV may be related with blocking 5-HT_3_R and downregulating SERT expression in the small intestine. In addition, the administration of *Rhus verniciflua* extract also has a beneficial effect of alleviating cisplatin-induced gastrointestinal inflammation via inhibited proinflammatory cytokines, like TNF-α, IL-1β, and IL-6, which may play an important role in chemotherapy-induced gastrointestinal side effects.

## 4 Conclusion and Outlook

CINV is still a troubling problem for patients who receive antitumor treatment. The underlying mechanism of CINV is still not completely clarified, and current studies suggest that the occurrence of CINV is mainly related to 5-HT/5-HT_3_R, SP/NK-1R, and DA/D_2_R signal pathways. As mentioned above, chemotherapy-induced gastrointestinal inflammation and gastrointestinal dysfunction also play a key role in CINV. At present, the mainstream therapeutic schemes of CINV are still dominated by the combination of 5-HT_3_R antagonist, NK-1R antagonist, and dexamethasone. Although this prophylaxis can effectively alleviate both acute and delayed CINV, the accompanying problems still need to be considered: 1) Will the adverse reactions of the antiemetic medicine increase the risk of chemotherapy and reduce the quality of life of cancer patients? 2) Will the high costs of antiemetics associated with the chemotherapy cycle become an additional financial burden for cancer patients? 3) Can we therefore find an ideal antiemetic drug that meets the requirements mentioned above? TCM might be one of the answers.

TCM, with more than 3,000 years of experience in treating diseases, have a promising future in the prevention and treatment of CINV. TCM has the advantages of having a relatively low price and multiple components and targets compared with antiemetic medicine. In this review, we introduce several TCMs that can be used to prevent and treat CINV (Table 2). Among them, a series of research have investigated the antiemetic effects as well as the mechanism of XBXD, ginger, and *Forsythiae Fructus*. The potential antiemetic mechanism of XBXD and ginger may be related to blocking 5-HT/5-HT_3_R, SP/NK-1R, and DA/D_2_R signal pathways peripherally and centrally. Also, XBXD and *Forsythiae Fructus* can mitigate gastrointestinal inflammation and regulate gastrointestinal dysfunction caused by chemotherapy agents. However, the evidence of other herbals against CINV mostly remains in the efficacy verification in animal models, and yet, the insufficient clinical data may affect its credibility. Therefore, further explorations on the mechanism of herbals against CINV are needed, and corresponding clinical observation may be carried out to clarify the antiemetic efficacy.

**TABLE 2:** The mechanism of TCM against CINV.

Traditional Chinese medicine	Main ingredients/Herbs	Animal models/Cell lines	Results	References
Xiao-Ban-Xia decoction	*Pinellia ternata*, *Zingiber officinale*	Cisplatin-induced vomiting model of minks	Reduces emesis (number of retching and vomiting) in minks within 72 h after cisplatin treatment	Qian et al. (2010a)
		Cisplatin-induced pica model of rats	Suppresses pica behaviors; regulates the synthesis and metabolism of 5-HT, SP, and DA; blocks the 5-HT_3_R, NK-1R, and D_2_R, both peripheral and central	Wang et al. (2010); Yu et al. (2015a); Yu et al. (2015b); Yu (2015); Li et al. (2020)
			Downregulates proinflammatory genes and modulates multiple inflammation-related signaling pathways; inhibits the overexpression of NF-κB and COX-2 and reduces plasma proinflammatory cytokine levels; suppresses the activation of NLRP3 inflammasome in the ileum	Li et al. (2017); Du et al. (2018); Li et al. (2020); Meng et al. (2020)
			Ameliorates cisplatin-induced abnormal gastrointestinal hormone expression, such as gastrin, motilin, somatostatin, and vasoactive peptide	Zhang et al. (2015)
		Cisplatin-induced delayed gastric emptying of mice	Promotes gastric emptying and intestinal movement	Liu et al. (2017)
Xiao-Ban-Xia-Fu-Ling decoction	*Pinellia ternata*, *Zingiber officinale*, *Poria cocos*	Cisplatin-induced pica model of rats	Inhibits the expression of SP and NK-1R in the medulla oblongata and gastric antrum	Zang et al. (2011)
Liu-Jun-Zi decoction	*Panax ginseng*, *Atractylodes macrocephala*, *Poria cocos*, *Glycyrrhiza glabra*, *Citrus* × *aurantium*, *Pinellia ternata*	Cisplatin-treated rats	Blocks 5-HT_3_R, 5-HT_2B_R, and 5-HT_2C_R; increases the circling ghrelin level	Takeda et al. (2008); Yakabi et al. (2010); Tominaga et al. (2011)
Ginger (*Zingiber officinale* Roscoe)	Ginger, 6-gingerol, 6-shogaol, and zingerone	5-HT-induced visceral afferent neuron response	Inhibits 5-HT_3_R	Jin et al. (2014)
	Gingerols	Cisplatin-induced vomiting model of minks	Reduces area postrema and ileum 5-HT, SP, and DA levels and inhibits ileum NK-1R expression	Qian et al. (2009); Qian et al. (2010b); Tian et al. (2020)
		Cisplatin-induced pica model of rats	Inhibits syntheses and promote the metabolisms of 5-HT, SP, and DA, and blocks the activations of corresponding receptors (5-HT_3_R, NK-1R and D_2_R) both centrally and peripherally	Qian et al. (2016); Tian et al. (2020)
	6-gingerol	Cisplatin-induced pica model of rats	Downregulates 5-HT synthesis, promotes 5-HT metabolism, and inhibits 5-HT_3_R expression; increases the relative abundance of Bacteroidetes and decreases Firmicutes on the phylum level; ameliorates abnormal gastrointestinal hormones and promotes gastric emptying	Feng et al. (2019); Cheng et al. (2020); Zhang et al. (2021)
*Forsythiae Fructus* (*Forsythia suspensa* (Thunb.) Vahl)	*Forsythiae Fructus* extract	Cisplatin-induced pica model of rats	Reduces plasma 5-HT level and blocks peripheral 5-HT_3_R; repairs damaged gastrointestinal mucosa; reduces plasma ROS, TNF-α, IL-1β, IL-18, and prostaglandin E2 levels; inhibits the overexpression of NF-κB, COX-2, and NLRP3 inflammasomes	Wang (2010); Meng et al. (2019); Meng et al. (2021)
		Cisplatin-induced delayed gastric emptying model of mice	Reduces the serum gastrin content and promotes small intestine movement; regulates gastrointestinal hormones gastrin, motilin, and somatostatin	Zuo (2015); Zhang et al. (2018)
	Forsythiaside A	Cisplatin-induced delayed gastric emptying model of mice	Regulates the gastrointestinal hormones as well as the activity of acetylcholinesterase and nitric oxide synthase activity in the gastric antrum and ileum	Bi et al. (2021)
Ginseng (*Panax ginseng* C.A. Mey)	Red ginseng extract	Cisplatin-induced vomiting model of ferrets	Reduces ferret vomiting and nausea in a dose-dependent manner	Kim et al. (2005)
	Korean ginseng root extract	Cisplatin-induced pica model of rats	Reduces kaolin consumption; saponin and non-saponin fraction may be the effective fractions of ginseng against CINV	Raghavendran et al. (2011); Sathyanath et al. (2013)
American ginseng (*Panax quinquefolius* L.)	American ginseng berry extract, ginsenoside Re	Cisplatin-induced pica model of rats	Reduces kaolin consumption; antioxidation effect	Mehendale et al. (2004); Mehendale et al. (2005)
	Ginseng saponins	Recombinant 5-HT_3A_R expressed in *Xenopus laevis* oocyte	Inhibits the peak current on 5-HT_3A_R induced by 5-HT, blocks the 5-HT_3A_R	Min et al. (2003)
*Scutellariae Radix* (*Scutellaria baicalensis* Georgi)	*Scutellariae Radix* extract	Cisplatin-induced pica model of rats	Reduces kaolin consumption	Aung et al. (2003); Mehendale et al. (2004)
*Ganoderma* (*Ganoderma lucidum* (Leyss. ExFr.) Karst.)	*Ganoderma* extract	Cisplatin-induced pica model of rats	Reduces kaolin consumption	Wang et al. (2005a); Yan (2008)
Grape seeds (*Vitis vinifera* L.)	Grape-seed extract	Cisplatin-induced pica model of rats	Reduces kaolin consumption; antioxidation effect	Wang et al. (2005b)
	Grape-seed proanthocyanin	Cisplatin-induced emesis model of pigeons	Reduces the vomiting bouts, retching rate, and weight loss rate; antioxidation effect	Alam et al. (2017)
*Rhus verniciflua* (*Toxicodendron vernicifluum* (Stokes) F.A. Barkley)	*Rhus verniciflua* extract	Cisplatin-induced pica model of rats	Reduces the consumption of kaolin in both the acute and delayed phases; blocks 5-HT_3_R and downregulates SERT expression in the small intestine; inhibits proinflammatory cytokines, like TNF-α, IL-1β, and IL-6	Kim et al. (2017)

It has been reported that the second-generation 5-HT_3_R antagonist palonosetron has promising effects on acute and delayed vomiting without combining with NK-1R (Wong et al., 1995; Saito et al., 2009). Further studies confirmed that the antiemetic effect of palonosetron is due to the crosstalk between 5-HT_3_R and NK-1R (Minami et al., 2001), therefore indicating that the crosstalk between 5-HT_3_R and NK-1R might be a novel mechanism in treating CINV. The NG108-15 cell line is currently known to express both 5-HT_3_R and NK-1R, which provides an ideal model for further study of the crosstalk between 5-HT_3_R and NK-1R (Emerit et al., 1993). So, the following interesting idea would come up: does this kind of signal crosstalk also exist in known antiemetic TCMs such as ginger, XBXD, or other herbal medicines? This question remains to be further explored. In addition, Borner et al. (2020b) and Breen et al. (2020) demonstrated that the activation of the GDF15/GFRAL signaling pathway by chemotherapy drugs may play an important role in nausea, vomiting, and anorexia. It provides us with a promising direction for further investigations on antiemetic herbal medicines against CINV based on the GDF15/GFRAL signaling pathway.

Despite the distinctive characteristics of TCM against CINV, it also has many problems that remain to be solved. Firstly, the active components of TCM, especially herbal formulas, are complicated, which limits the further elucidation of the potential mechanism of TCM against CINV. Therefore, future studies should focus more on clarifying the constituents of TCM, such as to establish the fingerprint of TCM and figure out the major antiemetic components so as to further investigate the antiemetic mechanisms. Secondly, there may be several different medicinal plant resources of the same herb, which makes the medicinal ingredients different and the pharmacological effects difficult to reproduce. So, an authoritative standard to regulate the source of TCMs are needed. Thirdly, studies published in the past decades mainly focused on verifying the antiemetic effect of TCM on different emetic animal models. It should be noted that there are species differences between animals and humans. Hence, more clinical trials are needed in future research, in order to comprehensively assess the efficacy of TCM against CINV.

Taken together, TCM have many advantages including a wide source, lower costs, and higher safety compared with the classic antiemetic medicines. On the basis of clarifying the active ingredients and combined with animal trials, clinical trials might be carried out in order to evaluate the feasibility of TCM against CINV.

## Abbreviations

5-HT, 5-hydroxytryptamine; 5-HT_2B_R, 5-hydroxytryptamine type 2B receptor; 5-HT_2C_R, 5-hydroxytryptamine type 2C receptor; 5-HT_3_R, 5-hydroxytryptamine type 3 receptor; 5-HT_3A_R, 5-hydroxytryptamine type 3A receptor; CINV, chemotherapy-induced nausea and vomiting; COX-2, cyclooxygenase-2; DA, dopamine; D_2_R, dopamine type 2 receptor; GDF-15, growth differentiation factor 15; GDNF, glial-derived neurotrophic factor; GFRAL, glial-derived neurotrophic factor family receptor α-like; IL-1β, interleukin 1 beta; IL-18, interleukin 18; IL-6, interleukin 6; LJZD, Liu-Jun-Zi decoction; MAO-A, monoamine oxidase A; NK-1R, neurokinin-1 receptor; NLRP3, NOD-like receptor family pyrin domain containing 3; NF-κB, nuclear factor-kappa B; ROS, reactive oxygen species; SERT, serotonin reuptake transporter; SP, substance P; TCM, traditional Chinese medicine; TPH, tryptophan hydroxylase; TNF-α, tumor necrosis factor alpha; TH, tyrosine hydroxylase; XBXD, Xiao-Ban-Xia decoction; XBFD, Xiao-Ban-Xia-Fu-Ling decoction
